# 3D Genome Engineering Using CRISPR/dCas Systems

**DOI:** 10.3390/ijms27188104

**Published:** 2026-09-11

**Authors:** Naida Yu. Mamaeva, Valeriy A. Yakovlev, Nikolay V. Kristovskiy, Pavel G. Feskin, Renat S. Vinnikov, Pavel D. Oleinikov, Sergey A. Shmakov, Konstantin V. Shaitan, Mikhail P. Kirpichnikov, Alexey K. Shaytan

**Affiliations:** 1 Department of Biology, Lomonosov Moscow State University, 119234 Moscow, Russiashaytan49@yandex.ru (K.V.S.); kirpichnikov@inbox.ru (M.P.K.); 2 Department of Fundamental Physical and Chemical Engineering, Lomonosov Moscow State University, 119234 Moscow, Russia; 3 Shemyakin-Ovchinnikov Institute of Bioorganic Chemistry, Russian Academy of Sciences, 117997 Moscow, Russia; 4 Institute of Gene Biology, 119334 Moscow, Russia

**Keywords:** 3D genome, chromatin loop, loop extrusion, epigenetics, epigenome editing, epigenetic engineering, CRISPR/Cas, dCas

## Abstract

The spatial organization of the genome has emerged as a central regulator of gene expression and cellular function. Chromatin architecture is organized hierarchically across multiple spatial scales and involves chromatin loops, topologically associating domains (TADs), chromatin compartments, and specialized nuclear environments that collectively shape regulatory interactions within the nucleus. Disruption of these structures contributes to a wide range of diseases, including developmental disorders, cancer, and laminopathies, stimulating growing interest in technologies capable of programmable manipulation of genome topology. The emergence of CRISPR/dCas-based technologies has transformed the field from descriptive 3D genomics to programmable genome engineering. Catalytically inactive Cas proteins fused to architectural or epigenetic effectors enable targeted manipulation of chromatin loops, loop extrusion, subnuclear positioning, and local chromatin states without altering the underlying DNA sequence. In this review, we summarize current CRISPR/dCas-based approaches for engineering three-dimensional genome architecture, discuss their mechanistic basis and applications, and highlight emerging therapeutic opportunities and major technical challenges in the field.

## 1. Introduction

**Figure 1:**
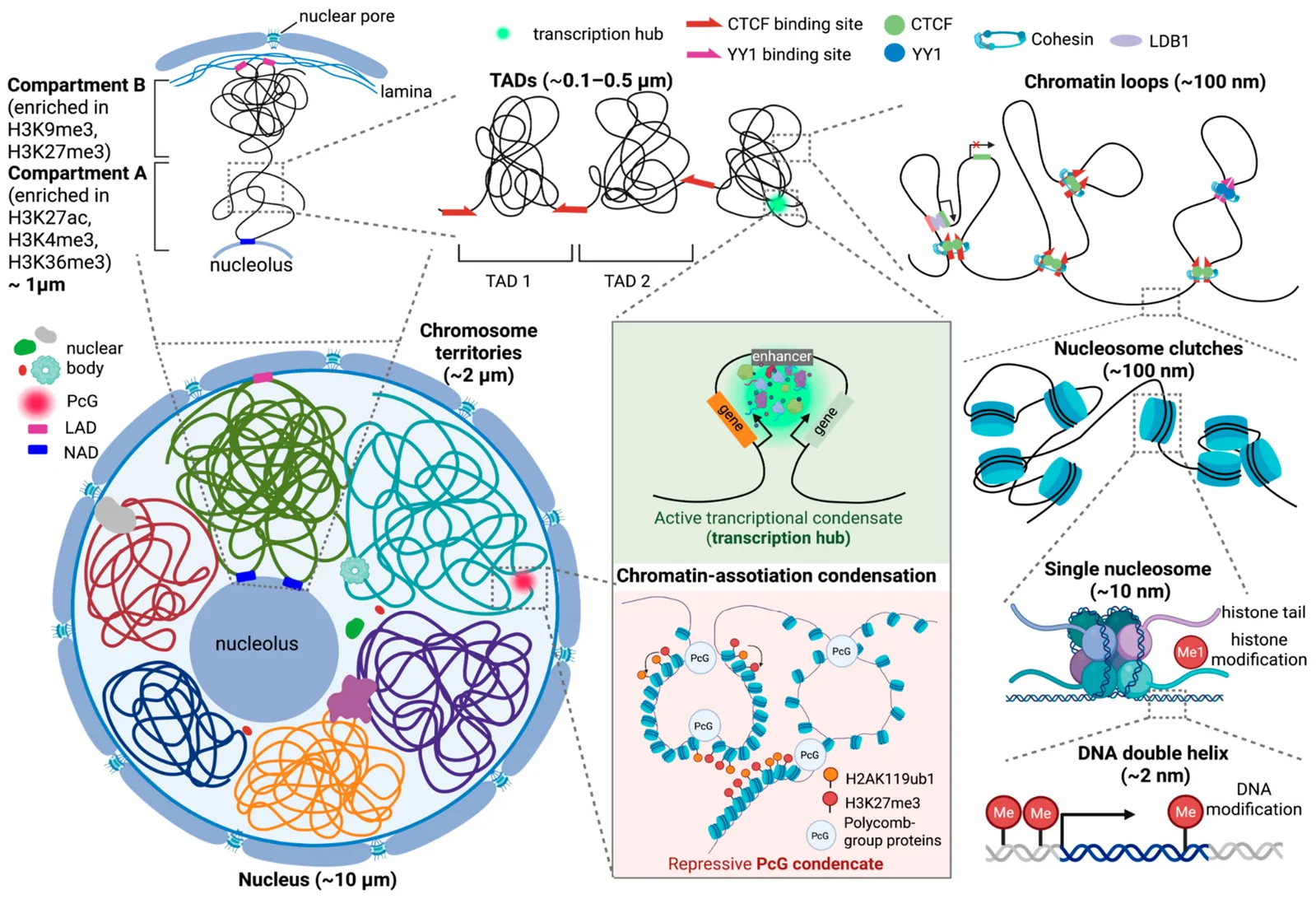
Current View of the Hierarchical Organization of the Genome. Genome organization spans chromosome territories, A/B compartments, topologically associating domains (TADs), chromatin loops, nucleosome clutches, nucleosomes, and DNA. Active chromatin can form transcription hubs, whereas Polycomb-repressed chromatin enriched in H3K27me3 and H2AK119ub1 can form Polycomb-group (PcG) condensates. Liquid–liquid phase separation (LLPS) may contribute to both. Approximate dimensions are indicated. LAD, lamina-associated domain; NAD, nucleolus-associated domain; CTCF, CCCTC-binding factor; YY1, Yin Yang 1; LDB1, LIM domain-binding protein 1. Histone marks follow standard nomenclature: ac, acetylation; me3, trimethylation; ub1, monoubiquitination. Created in BioRender. Shaytan, A. (2026) https://BioRender.com/rq1hva2.

Chromatin is currently understood not merely as a packaging form of genomic DNA, but as a dynamic regulatory system that integrates genome architecture with gene expression and cellular identity [1]. The mammalian genome exhibits hierarchical three-dimensional organization across multiple spatial scales, ranging from nucleosome-level chromatin folding to chromosome-scale compartmentalization (Figure 1) [2].

At the nanoscale level, nucleosomes assemble into heterogeneous and dynamic chromatin nanodomains, often referred to as nucleosome clutches, whose structural properties depend on histone modifications, linker histones, chromatin-binding proteins, and transcriptional activity [3]. This compositional heterogeneity also modulates intrinsic nucleosome dynamics: alterations in histone or DNA sequences, histone post-translational modifications, and the recruitment of chromatin proteins influence DNA processing during transcription, replication, and repair [4]. Linker histone H1 binds nucleosomal DNA near the dyad and contacts linker DNA at the nucleosomal entry–exit region, while its positively charged C-terminal domain promotes higher-order chromatin compaction [5]. These local chromatin states influence the mobility of genomic loci, accessibility of regulatory sequences, and the probability of transcription-factor binding [6,7,8,9].

At larger spatial scales, individual chromosomes occupy discrete chromosome territories within the interphase nucleus, whose radial positioning correlates with gene density, transcriptional activity, and chromatin state [10,11]. Chromatin further segregates into active (A) and inactive (B) compartments—a genome-wide separation that operates both within and between chromosome territories—reflecting the partitioning of transcriptionally permissive and repressed genomic regions [12]. A further level of organization is provided by topologically associating domains (TADs), which constrain enhancer–promoter communication and contribute to regulatory specificity [1,13,14]. Although subsets of TAD boundaries and compartment structures are conserved across cell types and species, chromatin interactions remain highly dynamic and undergo continuous remodeling during development, differentiation, and transcriptional activation [15,16].

Importantly, genome architecture combines structural stability with substantial plasticity. Rather than representing a static scaffold, chromatin organization is now viewed as an active and dynamic component of genome regulation. However, the extent to which specific architectural structures directly determine gene expression remains incompletely understood [17,18,19].

The prevailing framework for higher-order chromatin folding in mammalian interphase chromosomes is the loop-extrusion model, in which cohesin extrudes chromatin loops until halted by convergently oriented CTCF sites [20,21]. However, acute perturbation of cohesin, CTCF, WAPL, or YY1—which variously eliminates, redistributes, or extends TADs and architectural loops—has little immediate effect on most enhancer–promoter contacts or steady-state transcription [22]. Consistently, Region Capture Micro-C revealed nested enhancer–promoter interaction networks, termed microcompartments, that persist following cohesin depletion and likely arise through compartmentalization and multivalent interactions rather than loop extrusion alone. Active enhancers are enriched in transcription factors, coactivators, chromatin-remodeling complexes, and associated RNAs that collectively establish local regulatory environments. Among these components, the SWI/SNF (BAF) complex plays a central role in maintaining enhancer accessibility and lineage-specific chromatin states [23]. Recruitment of SWI/SNF complexes to cell-type-specific enhancers is mediated through combinatorial interactions with pioneer transcription factors and coactivators [24,25]. These observations further emphasize that enhancer function depends not only on linear DNA sequence but also on dynamic and spatially organized chromatin environments.

How enhancers transmit activating information to target promoters remains actively debated [26]. Several non-exclusive models have been proposed to explain enhancer function, including direct looping, chromatin-state spreading, facilitated tracking of regulatory factors, and transient transcriptional hubs enriched in coactivators and intrinsically disordered proteins [27]. Regulatory communication may instead be supported by dynamic, multivalent transcriptional hubs or condensate-like assemblies that transiently concentrate enhancers, promoters, transcription factors, and coactivators without requiring the formation of stable direct enhancer–promoter loops [28,29].

Live-cell imaging studies demonstrated that transcriptional activation can occur without stable enhancer–promoter proximity [30]. These findings are consistent with transcriptional bursting models in which enhancers primarily regulate burst frequency, whereas promoter architecture more strongly influences burst size [31]. Thus, chromatin architecture may influence the probability and kinetics of transcriptional activation rather than rigidly determining gene expression states.

Beyond chromosome folding itself, the eukaryotic nucleus is organized into specialized subnuclear compartments that contribute to genome compartmentalization and regulation. Large genomic regions associate with the nuclear lamina and nucleolus, forming lamina-associated domains (LADs) and nucleolus-associated domains (NADs), respectively. LADs are generally gene-poor, late-replicating heterochromatic regions tethered to the nuclear periphery by lamin-associated factors, whereas NADs are linked to the nucleolus and include both constitutive and developmentally regulated facultative heterochromatin, with recent studies distinguishing more stable LAD-overlapping regions from transcriptionally plastic NAD subsets [32,33].

In addition to these large domains, the nucleus contains dynamic membraneless compartments such as Cajal bodies, paraspeckles, and PML nuclear bodies, which spatially organize transcription, RNA processing, stress responses, and genome maintenance. PML nuclear bodies are SUMOylation-rich hubs involved in DNA damage response and antiviral defense, and can be reorganized by viruses such as adenovirus to facilitate replication and coupling to host transcriptional machinery [34]. Paraspeckles are built around the long noncoding RNA NEAT1 and function as regulatory hubs that sequester proteins to modulate gene expression under stress [35,36]. Many of these structures form through weak multivalent interactions involving intrinsically disordered proteins and RNA, often exhibiting properties consistent with liquid–liquid phase separation–like (LLPS) behavior [37,38]. LLPS provides a framework for understanding how such compartments organize chromatin and regulatory factors into dynamic assemblies, contributing to the formation of both repressive heterochromatin regions and transcriptionally active hubs in euchromatin [39,40,41]. Collectively, these organizational principles link nuclear architecture to transcriptional regulation, chromatin accessibility, and genome stability.

The growing recognition that three-dimensional genome organization plays a central role in gene regulation has stimulated intense interest in technologies capable of directly manipulating chromatin architecture. While chromosome conformation capture methods and advanced imaging approaches have substantially improved the understanding of genome topology, these approaches remain largely descriptive and do not allow direct interrogation of causality between chromatin structure and genome function.

The emergence of CRISPR/dCas-based systems has fundamentally transformed this landscape by enabling programmable and locus-specific manipulation of genome organization without altering the underlying DNA sequence [42,43,44,45,46]. Catalytically inactive Cas proteins fused to architectural, epigenetic, or condensate-forming effectors can be used to induce or disrupt chromatin loops, modulate cohesin-dependent loop extrusion, reposition genomic loci within nuclear compartments, and alter local chromatin environments. These architecture-focused approaches complement the broader field of dCas-based epigenome engineering, in which dCas proteins recruit transcriptional or epigenetic effectors to modify local chromatin states without changing the DNA sequence [47]. Three-dimensional genome engineering extends this principle to the spatial organization of regulatory elements and nuclear compartments, while epigenetic and topological changes can mutually reinforce one another. Together, these tools enable increasingly precise spatial, temporal, and combinatorial control of genome function.

These technologies provide powerful experimental platforms for dissecting the mechanistic relationships between chromatin architecture and transcriptional regulation while simultaneously opening new possibilities for synthetic biology, disease modeling, and therapeutic intervention. In this review, we consider 3D genome engineering as a continuum of programmable interventions ranging from direct physical manipulation of chromatin contacts and nuclear positioning to epigenetic perturbations that alter higher-order genome organization through architectural proteins, chromatin accessibility, or local chromatin state. Accordingly, epigenome-editing approaches are included when their effects extend beyond local transcriptional regulation and involve a measurable or mechanistically defined change in chromatin topology, insulation, compartmentalization, or spatial positioning. This perspective distinguishes the present review from works focused on CRISPR/dCas-based epigenome engineering, which primarily consider programmable modification of local chromatin states and transcriptional activity. Here, the emphasis is instead placed on the spatial dimension of genome regulation and on tools that directly manipulate, or indirectly reshape, higher-order chromatin organization. We consider a broad range of programmable approaches to 3D genome engineering, including both direct physical manipulation of chromatin contacts and nuclear positioning and indirect modulation of higher-order genome organization through architectural proteins, chromatin accessibility, or local chromatin state.

## 2. Precision Engineering of Chromatin Loops

**Figure 2:**
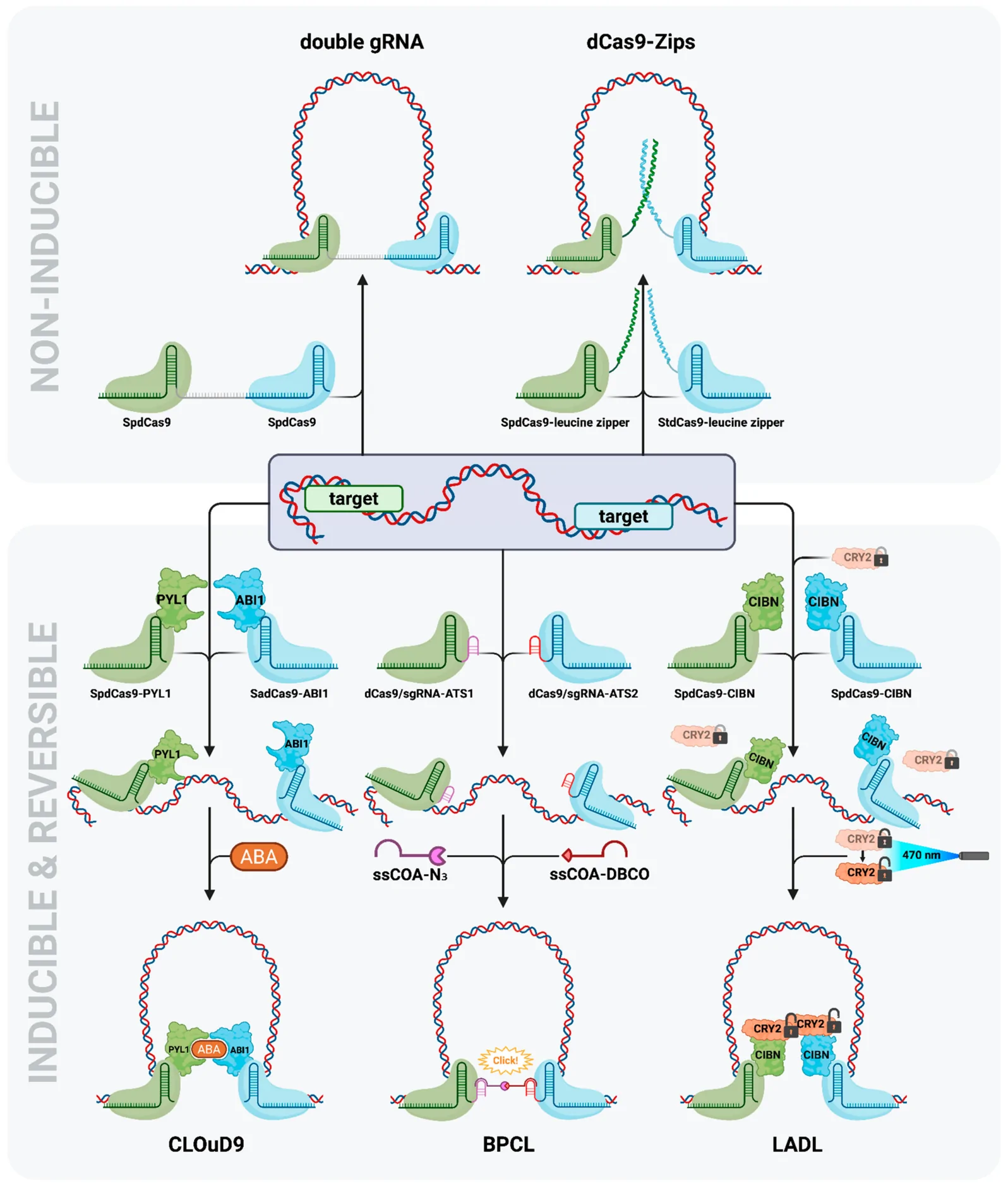
Overview of programmable CRISPR/dCas9-based tools for engineering chromatin loop formation. Schematic representation of constitutive (**top**) and inducible (**bottom**) molecular systems bridging distant genomic loci. dgRNA: a constitutive approach in which two guide RNAs are fused by a nucleotide linker within a single transcript, enabling two dSpCas9 proteins to simultaneously occupy two genomic targets. dCas9-Zips: a constitutive system in which heterodimeric leucine zipper domains fused to orthologous dSpCas9 and dStCas9 mediate post-translational protein–protein dimerization. CLOuD9: a chemically inducible system based on dSpCas9–PYL1 and dSaCas9–ABI1 heterodimerization triggered by abscisic acid (ABA) addition, and reversed upon its removal. BPCL: a bioorthogonal system in which two dCas9 complexes, guided by sgRNAs carrying adapter target sequences (ATS; short extensions appended to the sgRNA scaffold that serve as hybridization handles for exogenously delivered oligonucleotide adapters), are covalently bridged via SPAAC/SPIEDAC click chemistry through single-stranded clickable oligonucleotide adapters (ssCOAs). LADL: an optogenetic system in which blue light (470 nm) triggers CRY2–CIBN dimerization and CRY2 oligomerization to induce chromatin looping, reversible upon cessation of illumination. The closed padlock indicates inactive CRY2 before illumination, whereas the open padlock indicates light-activated CRY2 capable of interacting with CIBN. Created in BioRender. Shaytan, A. (2026) https://BioRender.com/z9yam8j.

The rational engineering of chromatin loops has evolved toward programmable and modular CRISPR/dCas-based platforms that facilitate genomic retargeting and, in some systems, enable inducible control and multiplexing (Figure 2). This evolution reflects a continuous effort to achieve greater precision, scalability, and temporal control over the 3D genome.

Early approaches to the rational engineering of chromatin loops relied heavily on programmable DNA-binding proteins. Pioneering studies utilized zinc finger proteins (ZFPs) fused to the self-association (SA) domain of the transcriptional cofactor LDB1, which naturally facilitates contacts between enhancers and promoters. This ZFP-SA strategy successfully established artificial loops between the enhancer (locus control region, LCR) and the promoter at the β-globin locus in erythroid cells, activating target gene transcription [48,49]. Notably, redirecting the LCR to the human γ-globin promoter raised γ-globin to approximately 85% of total β-like globin synthesis, with a reciprocal reduction in adult β-globin, demonstrating that a single engineered loop can dominate transcriptional output at a locus. Subsequently, the same self-association (SA) principle was ported to transcription activator–like effectors (the TALE-SA system): TALEs fused to the LDB1 dimerization SA domain and targeted to the γ-globin promoters drove forced reactivation of fetal globin in human umbilical cord blood–derived erythroid progenitor (HUDEP-2) cells, increasing γ-globin mRNA more than 70-fold with a concomitant reduction in β-globin and raising fetal globin to roughly 30% of total globin, mirroring the ZFP-SA outcome at the same locus [50].

However, while these approaches proved the functional significance of artificial looping, they presented a major bottleneck: systems based on ZFPs and TALEs require complex, custom protein design for each new target locus, making them exceedingly difficult to scale or multiplex.

The limitations of protein engineering were largely overcome with the advent of CRISPR/dCas9 platforms. One such static CRISPR tool is the dCas9-Zips (CRISPR-zippers) system. It utilizes two orthologs of the catalytically inactive nuclease dSpCas9 (Streptococcus pyogenes) and dStCas9 (Streptococcus thermophilus) fused to leucine zipper domains. Targeted to distant genomic regions via guide RNAs, the leucine zippers mediate post-translational heterodimerization to form a bivalent protein complex, inducing chromatin loop formation in *E. coli*; in a defined looping assay this reached approximately 15% efficiency for a 4.7 kb loop and was further improved by multiplexing additional guides, while rewiring a distal enhancer to its target promoter activated the endogenous *norVW* genes. Optimizing this strategy by fusing the two dCas9 orthologs into a single polypeptide eliminated the need for leucine zippers, but the resulting contacts remained static, precluding fine temporal control over their dynamics [42].

Seeking a more streamlined approach, researchers developed the conceptually compact double guide RNAs (dgRNA). In this minimalistic architecture, two sgRNA units are covalently linked within a single RNA molecule, allowing it to simultaneously recruit two dSpCas9 proteins to distinct genomic targets. Because loop formation requires only dCas9 and RNA, dgRNA is a minimal system that is highly attractive for size-constrained delivery vectors; in *E. coli* it reconstituted LacI-mediated LacZ repression at distal sites at levels comparable to conventional targeting. Nevertheless, its functionality in mammalian cells and its potential for reversible control require further development [46]. Because both systems target bacterial DNA rather than mammalian chromatin, their looping performance is not directly comparable to that of the mammalian platforms discussed below.

Inducible systems overcome the limitation of static contacts and provide capable of dynamic 3D genome reorganization in mammalian cells.

The CLOuD9 (Chromatin Loop Reorganization using CRISPR-dCas9) platform utilizes dSpCas9 and dSaCas9 fused to a PYL1 receptor and ABI1 phosphatase, respectively. The addition of the plant hormone abscisic acid (ABA) triggers their heterodimerization, physically pulling genomic loci together. Loops become detectable within 24 h of ABA addition and, crucially, CLOuD9 is fully reversible—removing ABA dissociates the complex—although prolonged induction (~10 days) stabilizes the contact into a washout-resistant state. The system has been used to reversibly activate endogenous transcription (for example, β-globin gene (*HBB*) in K562 cells and *POU5F1* (OCT4) in HEK293T cells), with the functional outcome being strongly context-dependent: the new LCR–β-globin loop activated transcription in K562 but not in HEK293T cells [43].

For even faster spatiotemporal resolution, LADL (Light-Activated Dynamic Looping) was developed. It employs a single dSpCas9 “anchor” fused to a CIBN domain alongside a soluble CRY2 photoreceptor “bridge”. Irradiation with blue light (470 nm) causes CRY2 to bind to CIBN and homo-oligomerize, pulling the targeted regions into a single cluster. This provides on-demand, de novo loop formation in as little as 4 h and instant reversibility when the light is removed. In mouse ESCs, however, a 2.0–2.5-fold increase in engineered loop strength produced only a modest 1.2–1.3-fold increase in target (*Zfp462*) expression, underscoring that contact frequency and transcriptional output are only loosely coupled [45].

Despite the success of dynamic systems, protein dimerizer-based platforms are inherently difficult to multiplex, as multiple pairs inevitably lead to stochastic crosstalk. The BPCL (Bioorthogonal Programmable Chromatin Looping) platform solves this through chemical orthogonality. In BPCL, guide RNAs carry adapter sequences that hybridize to single-stranded clickable oligonucleotide adapters (ssCOAs). These adapters utilize bioorthogonal click reactions (e.g., SPAAC and SPIEDAC) for in situ ligation, allowing the establishment of multiple isolated, selective chromatin contacts within a single cell. BPCL is therefore both inducible and reversible: adding the ssCOAs actively triggers loop formation, whereas UV irradiation cleaves the linker to disassemble the loop. Furthermore, BPCL can create multi-input hubs (connecting one promoter to two enhancers simultaneously) for synergistic transcriptional effects, juxtaposing pluripotency-gene promoters to their enhancers and activating their endogenous expression without cross-talk [44].

A comparison of these systems across key engineering parameters is provided in Supplementary Table S1, also available online as an interactive table at https://intbio.github.io/Mamaeva_et_al_2026/ST1 (accessed on 26 August 2026).

## 3. Tuning Chromatin Loop Extrusion Through dCas-Based Tools

### 3.1. Biological Basis of Loop Extrusion Engineering

The spatial folding of the mammalian genome at the nuclear scale is a multiscale and partially hierarchical process that contributes genome stability, transcriptional regulation, and cell differentiation [51]. During interphase, individual chromosomes occupy distinct three-dimensional regions known as chromosome territories (Figure 1). At the sub-chromosomal level, the genome is partitioned into A and B compartments, reflecting the spatial segregation of active (open) and repressed (closed) chromatin, respectively. Local interactions ranging from tens of kb to several Mb are organized into topologically associating domains (TADs), within which the frequency of spatial contacts is significantly higher than across their boundaries [52].

Accumulating evidence indicates that TADs and chromatin loops contribute to gene regulation by shaping the spatial landscape in which regulatory elements interact with their target genes. Numerous studies have shown that enhancer–promoter communication is strongly influenced by three-dimensional genome architecture, and that alterations in chromatin organization can lead to substantial changes in transcriptional activity without modifying the underlying DNA sequence itself [1,22,26,30,52]. These observations established genome topology as an important layer of gene regulation and motivated the development of approaches aimed at manipulating chromatin architecture in a programmable manner. Among the mechanisms proposed to organize chromatin in three dimensions, loop extrusion has emerged as the most widely supported framework for explaining the formation of chromatin loops and TADs, making it a particularly attractive target for genome engineering strategies [51,53].

### 3.2. Molecular Mechanisms Controlling Loop Extrusion

**Figure 3:**
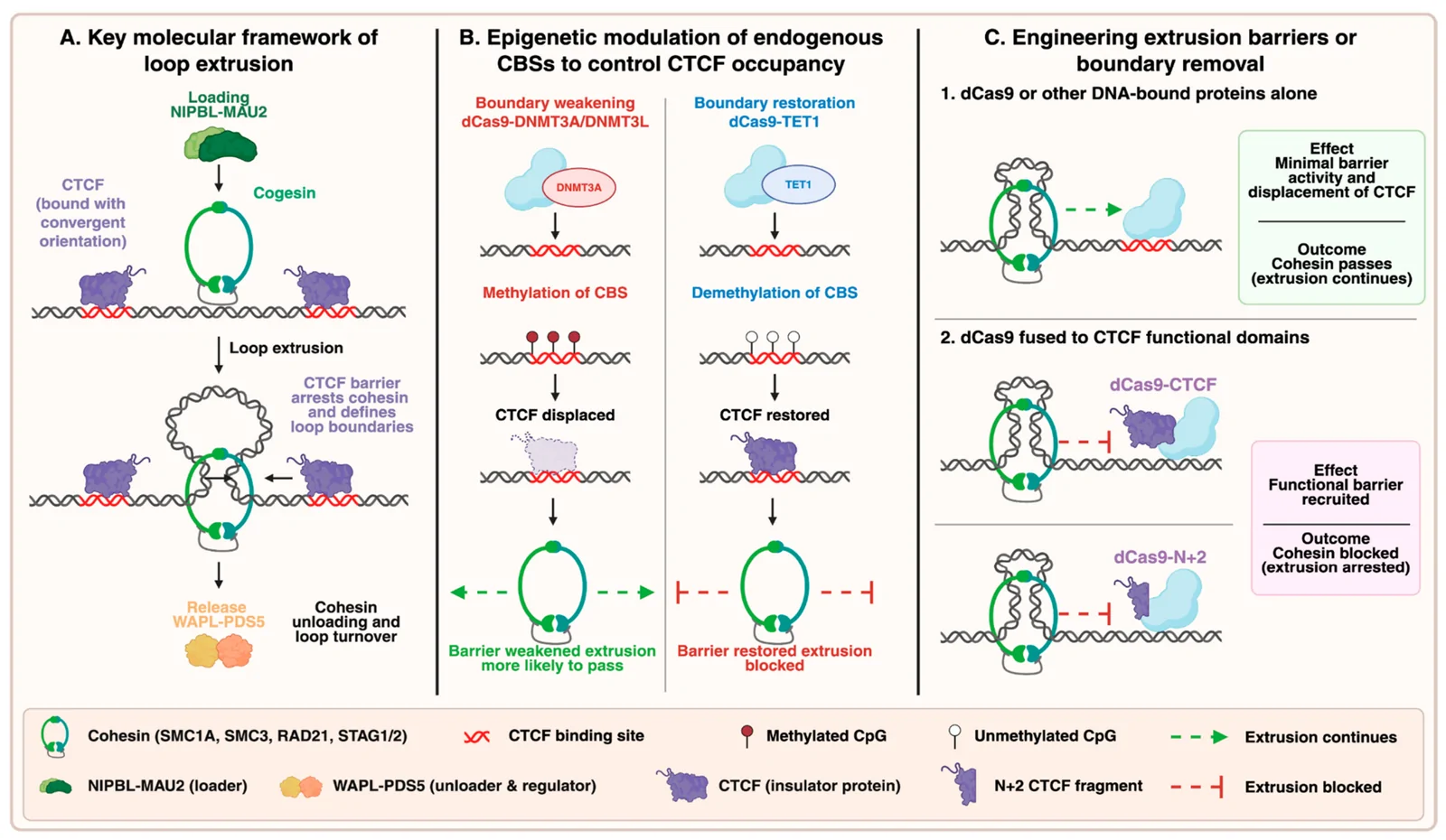
dCas-based modulation of cohesin-mediated loop extrusion and CTCF-dependent chromatin boundaries. (**A**) NIPBL–MAU2 promotes cohesin loading and loop extrusion, which is arrested by convergently oriented CTCF, whereas WAPL–PDS5 regulates cohesin release and loop turnover. (**B**) Targeted methylation of endogenous CTCF-binding sites (CBSs) by dCas9–DNMT3A/DNMT3L reduces CTCF occupancy and weakens boundary activity, while dCas9–TET1-mediated demethylation restores CTCF binding. (**C**) DNA-bound dCas9 can displace endogenous CTCF but provides only a weak extrusion barrier, whereas recruitment of full-length CTCF or its N + 2 fragment may provide partial barrier activity and impede cohesin-mediated extrusion. CTCF, CCCTC-binding factor; N + 2, CTCF N-terminal region with the first two zinc fingers; DNMT3A, DNA methyltransferase 3A; DNMT3L, DNA methyltransferase 3-like; TET1, ten-eleven translocation methylcytosine dioxygenase 1. Created in BioRender. Shaytan, A. (2026) https://BioRender.com/tpegaah.

Current models describe loop extrusion as a dynamic process driven primarily by the cohesin complex and regulated by boundary elements associated with CTCF [51]. Rather than functioning as a static architectural scaffold, cohesin continuously loads onto chromatin, translocates along DNA, extrudes loops, and is eventually released. The complex is organized around an SMC1-SMC3-RAD21 ring and operates together with several regulatory factors, including STAG1/2, the loading factor NIPBL-MAU2, and the unloading regulators WAPL and PDS5 [53,54]. The core molecular framework of cohesin loading, loop extrusion, CTCF-mediated arrest, and cohesin turnover is summarized in Figure 3A.

Loading of cohesin onto chromatin and initiation of loop extrusion are promoted by the NIPBL-MAU2 complex, which stimulates ATP-dependent conformational transitions within cohesin. Although the molecular mechanism of loop extrusion remains an active area of investigation, current structural and biochemical evidence is most consistent with a mechanistic framework commonly referred to as the hold-and-feed model [55,56]. In this framework, cohesin simultaneously maintains contact with an already extruded DNA segment while capturing a neighboring DNA region through ATP-dependent conformational changes. Cyclic transitions between distinct structural states progressively transfer newly captured DNA into the growing loop, resulting in directional loop enlargement. This model currently provides the most detailed description of cohesin-mediated extrusion and is broadly consistent with available experimental observations.

At the same time, several aspects of the extrusion cycle remain unresolved. In particular, the precise sequence of ATP-driven conformational transitions, the contribution of Brownian motion to DNA translocation, and the structural identities of key intermediate states remain incompletely characterized. Consequently, existing models should be regarded as mechanistic frameworks rather than definitive descriptions of cohesin function [57].

While cohesin acts as the motor of loop extrusion, the genomic positioning and stability of loop boundaries are primarily determined by the CCCTC-binding factor (CTCF) [58]. CTCF recognizes a directional DNA motif known as the CTCF binding site (CBS) through its central zinc-finger DNA-binding domain. Because the CBS consensus sequence is asymmetric, CTCF binds DNA with a defined orientation. As a result, cohesin encounters distinct molecular interfaces depending on the direction from which it approaches the bound CTCF molecule. Genome-wide analyses have demonstrated that stable chromatin loops preferentially form between convergently oriented CBSs, explaining the characteristic enrichment of convergent CTCF pairs at TAD boundaries [59].

Experimental evidence further indicates that the N-terminal region of CTCF plays a critical role in establishing directional barrier activity. In particular, a conserved YXF/YDF-like motif located within the N-terminus contributes to interactions that stabilize cohesin at chromatin boundaries and underlies the pronounced polarity of CTCF-mediated extrusion arrest [58]. Importantly, cohesin is not necessarily blocked by every protein bound to DNA. Instead, efficient boundary formation appears to depend on specific molecular interactions between cohesin and CTCF, helping explain why convergent CTCF sites represent uniquely robust extrusion barriers within mammalian genomes.

The lifetime and distribution of chromatin loops are additionally regulated by cohesin turnover factors. WAPL promotes cohesin release from chromatin, whereas PDS5 proteins modulate cohesin residence time and cooperate with WAPL in controlling extrusion dynamics. Together, these factors determine the balance between loop formation and loop dissolution, thereby influencing the overall architecture of chromatin domains [54].

In addition to CTCF-dependent boundaries, loop extrusion can be influenced by several context-dependent factors, including transcriptional activity, replication-associated protein assemblies, and local nucleic acid structures such as R-loops [60]. Although these elements may affect cohesin progression under specific conditions, their contribution appears substantially more variable than the central roles of cohesin, CTCF, and WAPL-mediated turnover.

### 3.3. Engineering Strategies Targeting Loop Extrusion

Because loop extrusion is controlled primarily through cohesin dynamics and CTCF-mediated boundary formation, most existing dCas-based chromatin engineering approaches target one of two processes: modulation of CTCF occupancy at endogenous binding sites or artificial installation of new extrusion barriers. Rather than directly altering DNA sequence, these approaches aim to modify the probability of chromatin contacts, the stability of looped states, or the strength of domain insulation.

One of the earliest demonstrations that chromatin topology could be manipulated through targeted epigenetic editing was provided by Liu et al. [61]. In this study, a dCas9-DNMT3A fusion was used to induce site-specific DNA methylation at endogenous CTCF binding sites. Targeted methylation reduced CTCF occupancy, weakened local chromatin interactions, and altered transcriptional activity within the affected domain. Importantly, these findings suggested that epigenetic modification of CBSs can indirectly reshape chromatin architecture without introducing permanent genetic alterations (Figure 3B, left).

Subsequent work expanded this concept by systematically comparing different strategies for disrupting CTCF occupancy. Using combinations of dCas9-KRAB and dCas9-DNMT3A/DNMT3L constructs, Amabile and colleagues demonstrated that efficient and persistent displacement of CTCF required precise targeting of the cognate CBS motif itself [62]. Methylation of neighboring regions was often insufficient to abolish CTCF binding, emphasizing that chromatin topology engineering depends strongly on guide positioning and local sequence context. Collectively, these studies established modulation of CTCF occupancy as one of the most direct approaches for controlling endogenous loop boundaries.

In addition to epigenetic editing, several studies have explored the use of dCas proteins as programmable competitors of CTCF binding. When targeted directly to CTCF binding sites, dCas9 can sterically interfere with endogenous CTCF occupancy, thereby weakening local insulation and increasing the probability that cohesin will traverse genomic regions that would otherwise function as extrusion boundaries. Unlike DNA methylation-based approaches, such perturbations are potentially reversible and do not require stable modification of the local epigenetic landscape. As a result, direct targeting of CBS provides a versatile strategy for investigating the contribution of individual boundaries to chromatin architecture and gene regulation.

Importantly, disruption of CTCF occupancy should not be confused with the creation of an alternative extrusion barrier. Recent single-molecule experiments demonstrated that DNA-bound dCas9 exhibits only limited ability to arrest cohesin translocation when compared with CTCF [58]. These observations indicate that the barrier function of CTCF cannot be explained solely by steric hindrance and likely depends on specific molecular features of the CTCF-cohesin interaction. Consequently, dCas9 appears to be more effective as a programmable tool for modulating endogenous CBS activity than as a direct substitute for CTCF-mediated boundary formation (Figure 3C, upper panel).

Several studies subsequently investigated whether targeted recruitment of CTCF could restore or establish chromatin contacts at selected genomic loci. A representative example was provided by Kubo and colleagues, who examined regulation of the *Vcan* locus during mouse embryonic stem cell differentiation [63]. Following deletion of an endogenous CBS, recruitment of CTCF through a dCas-based targeting system partially restored long-range chromatin interactions and increased transcriptional activity. Although the engineered contacts did not fully recapitulate the native chromatin architecture, these experiments supported the idea that targeted deployment of CTCF can influence local regulatory communication.

The molecular basis of such approaches is closely linked to the domain organization of CTCF itself. Detailed structure-function analyses demonstrated that different regions of CTCF contribute distinct activities, including DNA recognition, recruitment of architectural cofactors, and extrusion barrier formation. In particular, studies by Zhou et al. highlighted the importance of the central zinc-finger domain not only for DNA binding but also for the organization of insulator-associated protein complexes, including BRD2 [64]. These observations further emphasized that successful engineering of chromatin boundaries requires preservation of specific CTCF functions rather than simple tethering of a generic DNA-bound protein.

A major advance in this area was achieved through the development of minimalist CTCF-derived barriers. Tiukacheva and colleagues demonstrated that fusion of full-length CTCF to dCas platforms can result in undesirable interactions with endogenous genomic targets and widespread perturbation of chromatin organization [65]. To address this limitation, the authors developed the N + 2 construct, which contains the N-terminal region of CTCF together with the first two zinc fingers while lacking the DNA-binding module responsible for recognition of endogenous CBSs. This truncated variant retained the ability to influence cohesin dynamics when recruited by dCas while substantially reducing the risk of genome-wide off-target architectural effects (Figure 3C, lower panel). The study illustrates an important principle for future chromatin engineering efforts: functional modules involved in extrusion control can be separated from those responsible for endogenous DNA recognition.

Beyond local manipulation of individual loops, dCas-based platforms have increasingly been applied to broader epigenetic reprogramming strategies. One notable example is the H19/IGF2 imprinting control region, where targeted demethylation using dCas9-SunTag-TET1 increased CTCF occupancy and partially restored normal allele-specific regulation (Figure 3B, right) [66]. Related approaches have also been explored in models of Angelman syndrome and Prader–Willi syndrome, where targeted epigenome editing altered the regulatory landscape of imprinted loci and influenced disease-associated transcriptional programs [67,68]. Although these studies were not designed exclusively to manipulate loop extrusion, they illustrate how modulation of CTCF-dependent insulation can contribute to the correction of complex epigenetic defects.

The most sophisticated implementations of chromatin topology engineering currently combine multiple dCas-based tools within a single experimental framework. Qian et al. employed a dual strategy consisting of dCas9-TET1-mediated DNA demethylation together with targeted recruitment of CTCF using a dCpf1-based platform to reactivate the silenced *MECP2* allele on the inactive X chromosome [69]. In this system, epigenetic reactivation and architectural insulation were manipulated simultaneously, demonstrating that chromatin topology editing can be integrated with broader epigenome engineering approaches. While such studies remain proof-of-principle demonstrations, they highlight the increasing sophistication of programmable chromatin engineering technologies.

Collectively, available evidence indicates that dCas-based systems can influence loop extrusion through several complementary mechanisms, including modulation of CTCF occupancy, targeted alteration of DNA methylation states, steric competition at CBS motifs, and recruitment of CTCF-derived barrier modules. These approaches have established a causal relationship between engineered chromatin topology and transcriptional regulation across a growing number of experimental systems.

At the same time, several important challenges remain unresolved. The efficiency of chromatin topology editing is often highly dependent on genomic context, guide positioning, and cell type. Moreover, many engineered chromatin contacts appear probabilistic rather than constitutive, reflecting the inherently dynamic nature of loop extrusion. The long-term stability and heritability of artificially induced topological states also remain poorly understood. Future developments will therefore likely focus on improving targeting precision, reducing off-target architectural perturbations, developing reversible and tunable chromatin-engineering systems, and integrating topology editing with broader epigenetic regulatory platforms. Together, these advances may provide increasingly powerful tools for investigating genome organization and its contribution to gene regulation in development and disease.

While these individual studies demonstrate the feasibility of manipulating CTCF-dependent boundaries, a direct comparison reveals two distinct axes of trade-off that should guide tool selection. For boundary disruption, the primary axis is reversibility versus persistence: direct dCas9 steric interference offers a rapid, fully reversible means of acutely testing boundary function, whereas epigenetic editing via dCas9-DNMT3A or dCas9-KRAB/DNMT3A/DNMT3L installs a more durable, potentially heritable state of CTCF depletion, contingent on guides being positioned precisely at the core CBS motif rather than flanking sequence [61,62]. For boundary creation, the relevant trade-off shifts to construct footprint versus off-target risk: recruitment of full-length CTCF can generate robust ectopic contacts but risks engaging endogenous CBSs genome-wide, producing widespread architectural side effects [65]. The N + 2 fragment addresses this not by trading away potency, but by uncoupling CTCF’s barrier-forming activity from its native DNA-binding domain: the truncated construct retains the ability to influence cohesin dynamics while, by lacking a functional DBD, substantially reducing the risk of genome-wide off-target engagement [63,65]. This distinction reflects a single underlying principle: dCas9 alone can displace a boundary through steric competition but cannot substitute for one, since it lacks the specific molecular interface that allows CTCF to arrest cohesin [58]. Boundary engineering therefore requires either recruiting a genuine barrier-forming module (full-length CTCF or N + 2) or accepting that a disrupted boundary is not equivalent to an actively created one. Tool choice should accordingly follow the experimental question: transient perturbation favors steric interference, durable loss-of-function models favor epigenetic editing, and de novo insulation favors the minimalist N + 2 construct over full-length CTCF. These CTCF-boundary engineering strategies are summarized in Table 1. A broader comparison with other classes of 3D genome-engineering tools, including the assays that provide direct structural evidence for each, is provided in Table S1 (interactive version: https://intbio.github.io/Mamaeva_et_al_2026/ST1, accessed on 26 August 2026).

## 4. Subnuclear Compartment Tethering and Spatial Repositioning

Beyond the broad segregation of chromatin into transcriptionally active euchromatin and silent heterochromatin, the 3D genome is shaped by specialized subnuclear compartments that sequester specific genomic regions [70]. Thus, a significant portion of heterochromatin is localized near the nuclear periphery and the perinucleolar region, forming Lamina-Associated Domains (LADs) and Nucleolus-Associated Domains (NADs), respectively. LADs are typically dense, heterochromatic structures tethered to the nuclear lamina by architectural proteins such as LAP2β and the lamin B receptor. These domains comprise approximately 40% of the genome and are generally characterized by low gene density and late DNA replication. NADs represent chromatin associated with the nucleolus, the site of ribosome biogenesis. Recent mapping has revealed two distinct classes: Type I NADs, which overlap with LADs and display characteristics of constitutive heterochromatin, and Type II NADs, which do not overlap with LADs and behave more like facultative heterochromatin. Type II NADs are enriched with developmentally regulated genes and display higher transcriptional plasticity compared to their Type I counterparts [33]. There are other subnuclear compartments in the nucleus, such as PML nuclear bodies (PML-NBs) are dot-like structures and matrix-associated protein complexes that serve as hotspots for SUMOylation. They are implicated in DNA damage sensing, stress responses, and antiviral defense [34]. Other specialized compartments include paraspeckles, which are built upon long noncoding “architectural RNAs” (arcRNAs) like NEAT1 and function as molecular sponges to sequester regulatory proteins [35].

Therefore, the spatial organization of the eukaryotic genome is a critical determinant of nuclear events, including DNA replication, repair, and the coordination of gene expression [71]. The compartmentalized organization of the nucleus offers opportunities to regulate gene expression by repositioning genomic loci to specific subnuclear compartments. While previously shown methods based on non-CRISPR technologies have been used to manipulate genomic positioning, they are constrained by the need for large, repetitive sequence integrations that can perturb native chromatin structure [72,73]. To overcome these limitations, several programmable CRISPR/dCas9-based systems have been engineered to tether specific genomic loci to distinct subnuclear compartments.

### 4.1. CRISPR-PIN (Position in the Nucleus)

**Figure 4:**
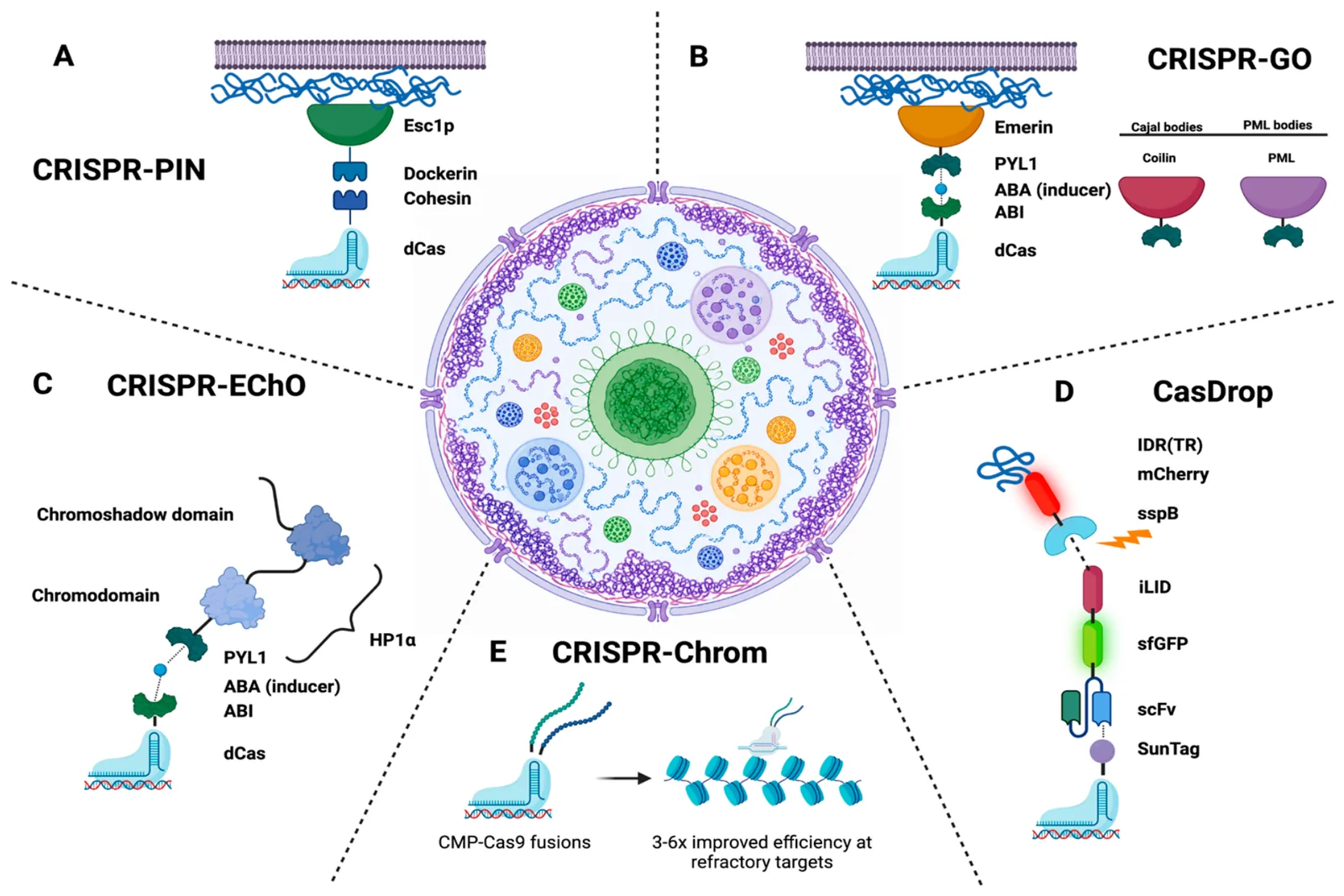
Overview of CRISPR/Cas-based systems for subnuclear repositioning, chromatin condensation, and modulation of Cas activity. (**A**) CRISPR-PIN uses the high-affinity interaction between a cohesin domain (Coh) fused to dCas9 and a dockerin domain (Doc) fused to the nuclear-envelope protein Esc1p to tether selected genomic loci to the nuclear periphery. (**B**) CRISPR-GO employs abscisic acid (ABA)-induced heterodimerization between dCas9–ABI and PYL1 fused to compartment-specific proteins, enabling inducible repositioning of genomic loci to the nuclear periphery, Cajal bodies, or PML nuclear bodies. (**C**) CRISPR-EChO uses ABA-inducible recruitment and spreading of HP1α-derived domains across dCas9-targeted repetitive regions, promoting reversible chromatin compaction, long-range interactions, and association with endogenous heterochromatin. (**D**) CasDrop combines a dCas9–SunTag targeting platform with light-inducible iLID–SspB interactions to recruit intrinsically disordered regions (IDRs) and nucleate local chromatin-associated condensates at selected genomic loci. (**E**) CRISPR-chrom employs Cas9 fused to CMPs derived from chromatin-associated proteins to increase DNA accessibility and improve editing efficiency at nucleosome-occluded or otherwise refractory genomic targets. ABA, abscisic acid; ABI, Abscisic Acid Insensitive; PYL1, pyrabactin resistance 1-like protein; HP1α, heterochromatin protein 1 alpha; PML, promyelocytic leukemia protein; IDR, intrinsically disordered region; CMP, chromatin-modulating peptide. Created in BioRender. Shaytan, A. (2026) https://BioRender.com/cz0s6g0.

Developed for the yeast *Saccharomyces cerevisiae*, CRISPR-PIN utilizes a synthetic protein scaffold based on the bacterial cohesin (Coh) and dockerin (Doc) protein interaction from *Clostridium thermocellum* [74]. In this system, *Streptococcus pyogenes* dCas9 [75] is fused to a bacterial cohesin domain, while a perinuclear membrane protein Esc1p is fused to a dockerin domain (Figure 4A) [76]. The tripartite interaction between the dCas9-Coh fusion, a guide RNA (gRNA), and the target DNA enables the programmable recruitment of native chromosomal loci to the nuclear periphery. Beyond reorganizing endogenous loci, CRISPR-PIN has been successfully applied to enable the controlled segregation of acentric plasmids, assisting the mitotic process by artificially tethering them to the nuclear envelope to ensure inheritance in daughter cells. It was also tested on relocalization of the chromosomal loci including *SCO2*, *ABP1*, *SAC7*, *HXK1*, and *IDI1* reaching up to 80% of the cells demonstrating target genes peripheral localization.

### 4.2. CRISPR-GO (CRISPR-Genome Organizer)

CRISPR-GO is a versatile system designed for human cells that provides inducible and reversible control over 3D genome organization (Figure 4B) [77]. This platform leverages chemically inducible heterodimerization, primarily the abscisic acid (ABA)-inducible ABI/PYL1 system. By fusing dCas9 to the ABI domain and compartment-specific proteins to the PYL1 domain, researchers can dynamically reposition genomic loci to the nuclear periphery via Emerin, to Cajal bodies via Coilin, or to PML bodies via PML. Cajal bodies contribute to snRNP assembly and maturation as well as telomerase RNP biogenesis [78], or PML bodies (via PML) [79]. Repositioning is rapid and mitosis-independent, allowing for the study of real-time dynamics. Notably, the colocalization of endogenous loci with Cajal bodies has been shown to significantly repress distal gene expression across linear distances of 30–600 kb. Such an approach allowed Wang et al. to reach up to 88% of the cells with induced relocalization [77].

### 4.3. CRISPR-EChO (CRISPR-Engineered Chromatin Organization)

While other systems focus on recruitment to existing bodies, CRISPR-EChO enables the inducible tiling of chromatin components across large linear regions spanning tens of kilobases (Figure 4C) [80]. Adapting the ABA-inducible platform [81], this system tiles human HP1α proteins across high-copy tandem repeats. The recruited HP1α molecules undergo homodimerization and oligomerization, forming a semi-stable polymer that reversibly transitions the targeted region from a diffuse to a compact state thereby tethering the targeted loci to a heterochromatin. This supranucleosomal-scale organization promotes long-range interactions in both cis and trans, integrates targeted loci into natural heterochromatin, and establishes a repressed transcriptional state that persists across over 600 kb.

It is important to note that a crucial role in the development of described tools belongs to the interaction between the designed agents with the chromatin-associated proteins in different subnuclear compartments. A revolutionary advancement in predicting and designing these spatial interactions is ProtGPS [82]. ProtGPS identifies a previously unrecognized “localization code” within protein amino acid sequences, allowing it to predict with 83% to 95% accuracy where a protein will localize among 12 different cellular compartments, including the nucleus, nucleolus, and nuclear speckles. For 3D genome engineering, ProtGPS offers a transformative capability: a generative algorithm that can design entirely novel amino acid sequences intended to localize to specific subnuclear environments. Experimental validation has demonstrated that these synthetic proteins can selectively self-assemble within desired compartments, such as the nucleolus, providing an innovative toolkit for engineered subnuclear tethering. Furthermore, ProtGPS can predict how pathogenic mutations alter this localization code, revealing mis-localization as a potentially underappreciated mechanism for various genetic disorders. Integrating ProtGPS with CRISPR/dCas9 platforms allows researchers to not only “drag” DNA to a compartment but to predict natural localization patterns and design the proteins that facilitate recruitment with high precision, ultimately advancing the design of targeted therapies for complex diseases [82]. The three tethering platforms are compared with each other, and with the looping and condensate-based systems, in Table S1, which also gives the model system and reported repositioning efficiency of each (interactive version: https://intbio.github.io/Mamaeva_et_al_2026/ST1, accessed on 26 August 2026).

## 5. Tuning dCas-Tools Activity Through Interactions with 3D Genome Architecture

Interactions between CRISPR/Cas systems and 3D genome architecture are bidirectional. On the one hand, nucleosome occupancy and chromatin accessibility influence target recognition and can limit CRISPR activity by rendering certain genomic sites refractory to modification [83]. On the other hand, programmable Cas platforms can be equipped with chromatin-modulating or condensation-promoting modules that reshape the local chromatin environment. CRISPR-Chrom and CasDrop illustrate these complementary strategies: CRISPR-Chrom enhances Cas activity at poorly accessible loci, whereas CasDrop uses optogenetically induced condensates to reorganize chromatin at selected genomic sites.

### 5.1. CRISPR-Chrom

To overcome these physical barriers, the CRISPR-chrom strategy fuses Cas9 with chromatin-modulating peptides (CMPs) derived from proteins like HMGN1 and HMGB1 (Figure 4E) [84,85]. These CMPs actively modify the local environment—HMGN1 promotes chromatin decompaction, while HMGB1 provides DNA bending activity—to facilitate tool access. Fusions such as HN1HB1 have demonstrated a three- to sixfold increase in efficiency at previously difficult targets. This method is highly versatile across diverse Cas9 orthologs, providing a robust way to tune tool activity by directly interacting with the dense 3D chromatin landscape. In addition to HMGN1 and HMGB1, the CRISPR-chrom strategy evaluated 20 different peptides known to interact naturally with chromatin. Successful fusions included the central globular domain of human histone H1, which competes for chromatin binding, and the DNA–binding domains of yeast remodeling complexes ISWI and CHD1. While full-length HMG proteins like HMGN2 through HMGN5 also improved activity, some modifications were counterproductive; for instance, fusions with the DNA–binding domains of human TOP1 or *Mycobacterium leprae* DNA helicase actually reduced editing efficiency. The most robust multi-peptide combinations identified—HN1H1G (HMGN1 and Histone H1) and HN1CHD1 (HMGN1 and CHD1)—provide a versatile toolkit for enhancing Cas9 performance across diverse and difficult-to-reach genomic targets [86].

### 5.2. CasDrop

CasDrop is an optogenetic technology that utilizes liquid–liquid phase separation (LLPS) to restructure the genome (Figure 4D) [87]. The system is modular, involving a dCas9-SunTag (ST) scaffold that recruits scFv-sfGFP-iLID. Upon blue light activation, the optogenetic protein iLID [88] binds to SspB fused to an intrinsically disordered region (IDR), such as those from BRD4, FUS, or TAF15, concentrating these IDRs at the target locus to drive localized condensation. These synthetic liquid droplets act as mechano-active chromatin filters [89]. Because growth is thermodynamically unfavorable in stiff environments, droplets preferentially nucleate in low-density euchromatin and mechanically exclude non-targeted chromatin. Conversely, surface tension-driven coalescence of droplets seeded at distal sites can pull targeted genomic elements together, generating forces estimated at 0.1–1 pN. Table S1 summarizes both systems together with the other CRISPR/dCas platforms discussed above, including the components each requires and the readouts on which its reported performance rests (interactive version: https://intbio.github.io/Mamaeva_et_al_2026/ST1, accessed on 26 August 2026).

## 6. Numerical Modeling and Physical Principles in Understanding the Function of 3D Genome Engineering Tools

Computational descriptions relevant to chromatin engineering span multiple spatial scales, from atomistic simulations of nucleosome dynamics to polymer and statistical-mechanical models of long-range DNA contacts. At the nucleosome scale, atomistic simulations indicate that the intrinsic bending plasticity of H2A–H2B dimers facilitates nucleosome sliding and affects DNA rewrapping kinetics, suggesting that histone variants and post-translational modifications may regulate chromatin structure through changes in dimer dynamics [90]. Among the currently available CRISPR/dCas-based approaches to 3D genome engineering, programmable loop-forming systems have so far been described most extensively using quantitative statistical-mechanical models. This section therefore uses loop-forming systems to illustrate how physical principles and mathematical modeling can guide the design and optimization of 3D genome engineering tools.

**Figure 5:**
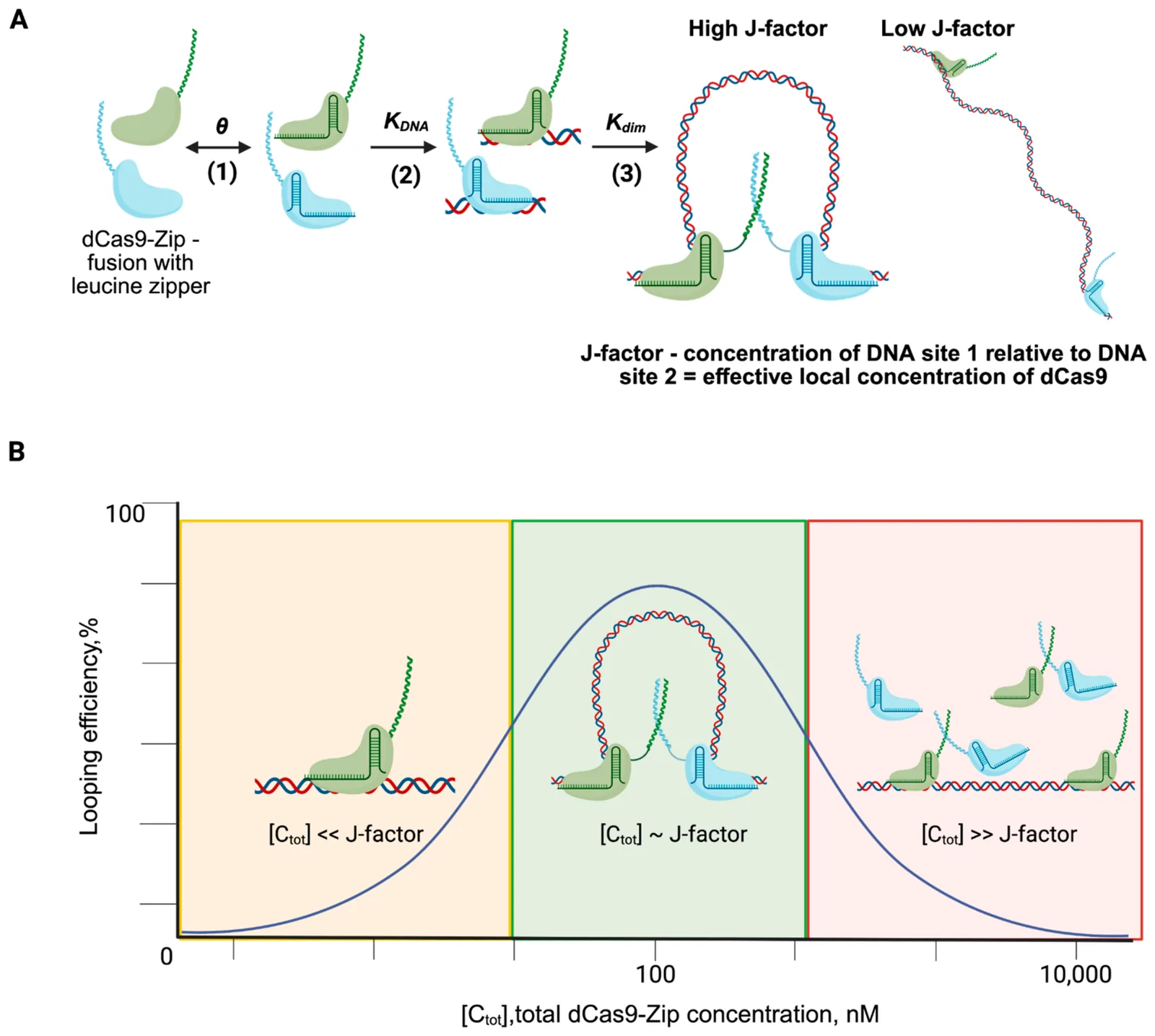
Physical principles of DNA loop formation using dCas-based systems: the dCas9–Zip system as an example. (**A**)—Reaction scheme with the definition of main modelling parameters [42] (**1**)—gRNA-dCas9-Zip complex formation, described by *θ* (fractional saturation of dCas9-Zip with gRNA) in the model, (**2**)—gRNA-dCas9-Zip DNA binding, described by *K_DNA_* (equilibrium dissociation constant) in the model (**3**). (**B**)—The looping efficiency (the fraction of time that the DNA loop is formed) as a function of the value of total dCas-Zip concentration with fixed values of other parameters. At first, dCas global concentration is too low to form dimers and loops. As the dCas global concentration reaches the value around the same order of magnitude as the local, frequency of loop formation increases, afterwards as dCas global concentration continues to grow, dCas local concentration becomes less than the global, which leads to the decrease in efficiency of loop formation. Adapted from [42]. Created in BioRender. Shaytan, A. (2026) https://BioRender.com/o8kwq36.

The design of loop-forming complexes should take into account several parameters: the number of targeted DNA-binding sites, the distance between them along the DNA, the affinity of protein–DNA binding, the affinity of protein–protein interactions (dimerization), the fractional saturation of dCas9 with sgRNA, and the total protein concentration. The spatial contribution of DNA tethering should also be considered. When a protein complex is bound to one target site, the second site has an effective local concentration relative to the first; this parameter is commonly referred to as the J-factor or jLOOP [42] (Figure 5A). Because all these parameters influence loop formation, quantitative models can help predict looping efficiency and identify optimal experimental conditions.

Hao et al. used a simple statistical-mechanical model to predict the efficiency of loop formation by dCas9-Zip dimers in *E. coli* cells [42]. The model includes different states of the system: free DNA, different unlooped but protein-occupied states, and looped DNA. Each state is assigned a statistical weight, which is then expressed in terms of the relevant equilibrium constants and the J-factor. All weights are normalized to that of the unoccupied DNA state [42,91].

The model developed by Hao et al. [42] also describes how looping efficiency depends on these parameters. As expected, increasing the fractional saturation of dCas9 with sgRNA increases looping efficiency. Decreasing either the dimer dissociation constant K_dim_ or the dissociation constant of the dCas9–sgRNA complex from DNA K_DNA_ favors loop formation [42]. However, the effect of the total dCas9-Zip concentration [C_tot_] is more complex. Under the simplifying assumptions of complete dimerization and saturation with sgRNA, loop formation is maximal when [Ctot] = K_DNA_ [42,92]. Consequently, the dependence of looping efficiency on [C_tot_] is bell-shaped for dCas9-Zip [42]. This behavior can be understood by considering the analysis of Priest et al., who compared two proteins with markedly different multimerization properties: LacI, which readily forms loop-competent tetramers in solution, and the λ CI protein, whose loop-stabilizing octamer assembles primarily through interactions between DNA-bound oligomers [92]. At high LacI concentrations, separate tetramers can occupy the two DNA-binding sites, preventing a single tetramer from bridging them and thereby reducing looping efficiency. As the distance between the DNA sites increases and the J-factor decreases, the protein concentration range compatible with efficient looping shifts toward lower values, which may be difficult to maintain reproducibly because of the stochastic nature of gene expression [92]. By contrast, λ CI-mediated looping becomes more efficient as the CI concentration increases. Even when the J-factor is low, the free energy of multimerization can compensate for the unfavorable energetic and entropic cost of loop formation [92]. The dCas9-Zip system resembles the strongly dimerizing LacI case because its low K_dim_ reflects strong protein–protein interactions. Increasing the dCas9-Zip concentration initially promotes target-site occupancy and loop formation. At sufficiently high concentrations, however, separate dCas9-Zip dimers increasingly occupy the two target sites and compete with productive bridging. In the model, looping is favored when the J-factor exceeds the concentration of free dCas9-Zip dimers in solution [92] (Figure 5B). The model developed by Hao et al. therefore allows optimal combinations of system parameters to be identified for improving looping efficiency [42].

A central parameter in these models is the J-factor, which, as noted above, depends on the distance between two sites along the DNA but also varies with chromatin structure. Rippe derived equations based on polymer models, including the freely jointed chain model, for calculating contact probabilities and effective local concentrations in different nucleic acid polymers, including interphase chromatin fibers [93]. Kharerin et al. subsequently calculated the J-factor for nucleosome-containing chromatin using a freely rotating chain model [94]. To our knowledge, quantitative models specifically describing dCas-mediated DNA looping in eukaryotic cells have not yet been developed. Such models could potentially be constructed by combining the equilibrium framework described above with existing polymer models of chromatin. Models intended for eukaryotic systems should also account for cohesin-mediated loop extrusion because DNA-bound Cas ribonucleoproteins and R-loops can act as barriers to cohesin translocation and thereby alter loop-extrusion dynamics [60]. These interactions could affect the stability and efficiency of engineered loops, with the magnitude of the effect likely depending on loop size and the positions of the engineered sites relative to endogenous extrusion domains.

Equilibrium statistical-mechanical models do not directly describe the timescales or dynamics of loop formation. More broadly, rational design of dCas-based genetic circuits requires quantitative characterization of component concentrations, DNA-binding affinities, off-target sequestration, and input–output transfer functions [95]. An important direction for future research is therefore the development of kinetic models of these systems. The related field of synthetic gene circuits has already made progress in kinetic modeling of dCas9-mediated regulation, and these approaches could potentially be adapted to loop-forming complexes [96,97,98].

These modeling approaches could provide a practical framework for the rational design of 3D genome-engineering experiments. For example, candidate gRNA pairs could be ranked according to the predicted contact probability or J-factor between their target sites, allowing guide selection to incorporate not only sequence specificity but also the spatial compatibility of the targeted loci. Polymer and loop-extrusion models could further guide the choice of target distance and positioning relative to endogenous CTCF/cohesin boundaries. Statistical-mechanical models could then be used to optimize protein concentration, DNA-binding affinity, dimerization strength, and component stoichiometry to maximize productive bridging while minimizing independent saturation of the two target sites [42]. Finally, machine-learning approaches could assist protein-domain design; for example, ProtGPS can predict and generate protein sequences with defined subcellular localization properties and could therefore facilitate the design of synthetic domains for targeting engineered loci to specific nuclear compartments [82].

## 7. Disease Modeling and Therapeutic Opportunities

The ability to manipulate chromatin architecture, enhancer–promoter communication, and subnuclear positioning has transformed CRISPR/dCas-based systems from tools for studying genome organization into promising platforms for disease modeling and therapeutic intervention. Many human diseases are associated not only with mutations in coding sequences but also with alterations in chromatin topology, enhancer activity, nuclear positioning, or epigenetic regulation [99,100]. Consequently, programmable manipulation of three-dimensional genome organization offers an opportunity to address disease mechanisms at the level of gene regulation rather than DNA sequence itself.

The therapeutic potential of programmable chromatin reorganization has already been demonstrated in several disease-relevant cellular models, including Rett syndrome, viral infection, and regulation of the β-globin locus. Although translation of these approaches to in vivo settings remains at an early stage, these studies establish proof of principle that targeted manipulation of genome architecture can modulate disease-associated gene regulation and provide a basis for future therapeutic development.

**Figure 6:**
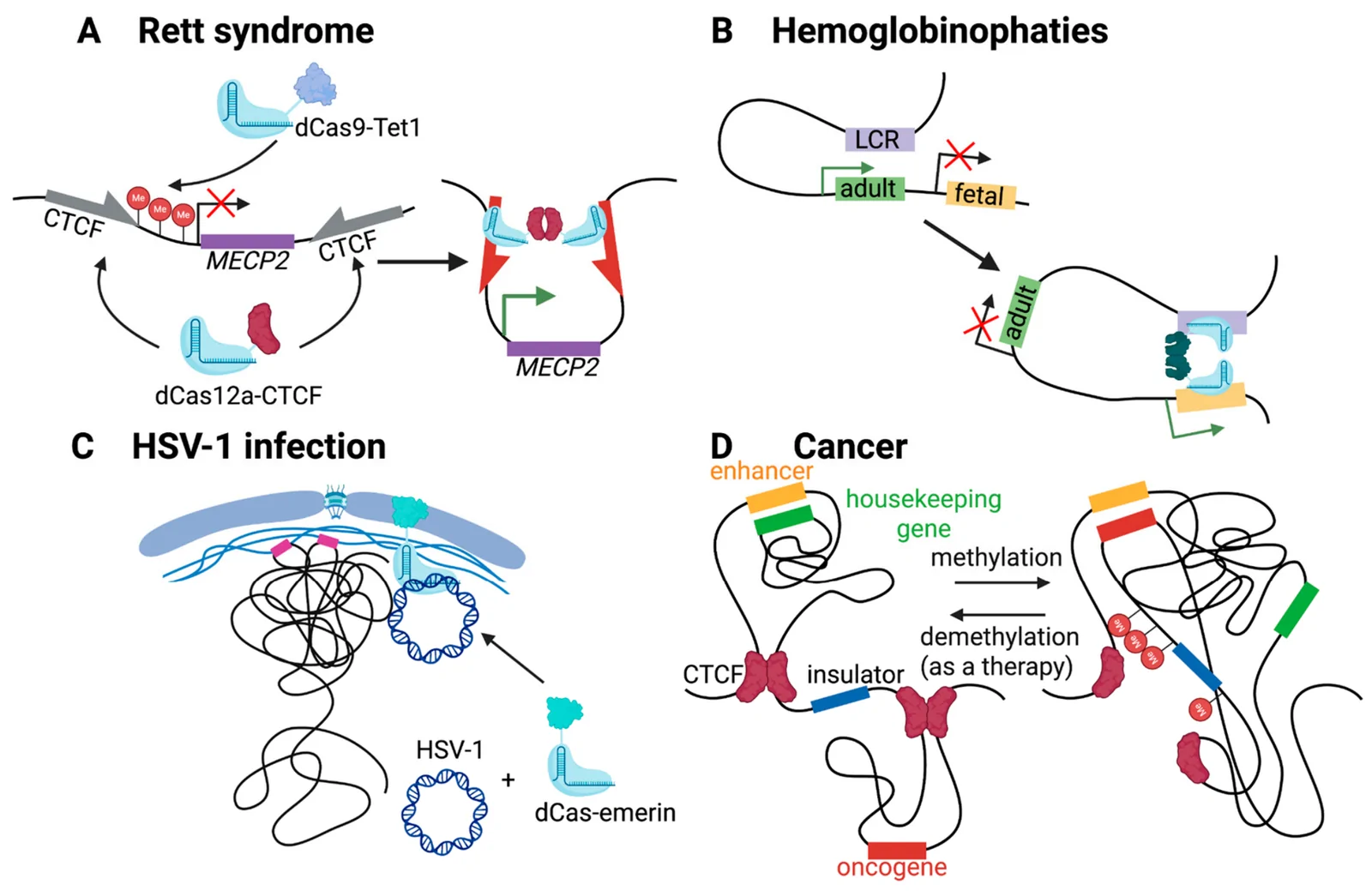
Therapeutic applications of CRISPR/dCas-mediated engineering of 3D genome organization. (**A**) Combined dCas9–TET1-mediated demethylation and targeted recruitment of CTCF using dCas12a can restore local chromatin architecture and enhance reactivation of the silenced *MECP2* allele in Rett syndrome [69]. (**B**) Programmable looping between the locus control region (LCR) and globin promoters may redirect globin expression for the treatment of hemoglobinopathies [43]. (**C**) dCas9–emerin-mediated repositioning of HSV-1 genomes to the nuclear periphery promotes a repressive chromatin environment and inhibits viral transcription and replication [101]. (**D**) Methylation-dependent loss of CTCF occupancy can disrupt TAD boundaries, permit enhancer hijacking, and activate oncogenes. Targeted demethylation or CTCF recruitment may restore insulation and prevent pathogenic enhancer–promoter communication [65,102]. Created in BioRender. Shaytan, A. (2026) https://BioRender.com/rrxopma.

One of the most compelling examples is provided by Rett syndrome, an X-linked neurodevelopmental disorder caused by loss-of-function mutations in *MECP2*. Because female patients retain a functional but transcriptionally silenced copy of *MECP2* on the inactive X chromosome, restoration of endogenous gene expression represents an attractive therapeutic strategy. Qian and colleagues observed increased CTCF occupancy at two chromatin anchor sites flanking the *MECP2* locus following successful epigenetic reactivation of the gene (Figure 6A) [69]. Based on this observation, the authors developed a dCpf1-CTCF platform to direct CTCF recruitment to these sites and recreate the associated chromatin architecture. When combined with targeted promoter demethylation using dCas9-TET1, engineered CTCF recruitment significantly enhanced reactivation of the silent *MECP2* allele compared with DNA methylation editing alone and resulted in improved rescue of Rett syndrome-associated neuronal defects. This work illustrates how targeted manipulation of local chromatin architecture can synergize with epigenetic editing to achieve more efficient therapeutic gene reactivation.

Hemoglobinopathies, including β-thalassemia and sickle cell disease, represent another potential application of 3D genome engineering (Figure 6B). Proof-of-concept studies at the β-globin locus demonstrated that programmable chromatin looping can activate endogenous globin gene expression and establish stable regulatory states, highlighting the therapeutic potential of chromatin architecture engineering for diseases caused by dysregulated gene expression [43].

Three-dimensional genome engineering is also emerging as a valuable platform for studying host–virus interactions. Many DNA viruses exploit nuclear architecture and chromatin organization during infection, yet distinguishing correlation from causation has historically been challenging [103]. To address this limitation, Xiang and colleagues developed CRISPR-nuPin, a dCas9–emerin-based system capable of rapidly repositioning extrachromosomal DNA to the nuclear periphery (Figure 6C). Application of this approach to herpes simplex virus type 1 (HSV-1) demonstrated that viral genomes artificially tethered to the nuclear lamina acquired repressive chromatin features, exhibited reduced transcriptional activity, and showed markedly impaired replication [101]. Conversely, repositioning viral genomes at later stages of infection promoted viral gene expression and replication, revealing a direct causal relationship between subnuclear localization and viral genome activity. These findings highlight programmable nuclear repositioning as a powerful strategy for dissecting host–virus interactions and suggest that manipulation of nuclear architecture may eventually provide novel antiviral therapeutic opportunities.

Beyond currently demonstrated proof-of-concept applications, three-dimensional genome engineering may provide new therapeutic opportunities in diseases driven by aberrant chromatin topology and enhancer–promoter communication. Cancer represents one of the most promising areas for future development. Numerous malignancies are associated with structural variants that rewire chromatin interactions, disrupt TAD boundaries, or promote oncogene activation through enhancer hijacking. Examples include activation of *MYC*, *TAL1*, *GFI1*, *LMO2*, and other oncogenes following the formation of aberrant enhancer–promoter contacts [104,105,106,107].

In addition to direct recruitment of architectural proteins, dCas-based systems can be used to modulate chromatin topology indirectly through targeted epigenetic editing of CTCF-binding sites [61]. Because CTCF occupancy is highly sensitive to local CpG methylation, programmable recruitment of DNA methyltransferases such as dCas9-DNMT3A enables selective methylation of CTCF anchor sites, resulting in loss of CTCF binding and disruption of CTCF-dependent chromatin loops. Experimental studies demonstrated that targeted methylation of individual CTCF loop anchors is sufficient to interfere with chromatin looping and alter gene expression within neighboring domains. A well-characterized example of the pathological consequences of such architectural disruption is provided by IDH-mutant gliomas, in which hypermethylation of CTCF-binding sites weakens TAD insulation and permits ectopic enhancer–promoter interactions, resulting in aberrant activation of *PDGFRA* [102]. These observations suggest that programmable epigenetic editing of architectural elements may ultimately provide a means to selectively disrupt or restore pathogenic chromatin interactions without introducing permanent genetic alterations. These applications are summarized in Table 2; the tools underlying them are compared with the full set of CRISPR/dCas platforms, including their experimental models and principal limitations, in Table S1 (interactive version: https://intbio.github.io/Mamaeva_et_al_2026/ST1, accessed on 26 August 2026).

Several disease-oriented applications of 3D genome engineering currently remain proof-of-concept studies performed in cultured cells, and their translation to in vivo settings will require overcoming several challenges. Some of these limitations are shared with other CRISPR/dCas-based technologies, including efficient and cell-type-specific delivery, immunogenicity, and control over the duration of the induced regulatory state [47]. These requirements are likely to be application-dependent: transient and reversible modulation may be sufficient or even preferable in some settings, whereas correction of chronic regulatory defects may require more persistent effects. Delivery is particularly relevant for multicomponent systems requiring large dCas fusion proteins, multiple guide RNAs, or simultaneous expression of interacting effectors. Therapeutic development will also require careful assessment of off-target and unintended transcriptional effects, especially when long-term or repeated interventions are considered [47]. Beyond these general limitations, manipulation of three-dimensional genome organization raises additional challenges related to genomic and cellular context, control of productive chromatin interactions, their dynamic nature, and the spatial extent of downstream architectural and transcriptional effects; these issues are discussed in detail in the following section.

Collectively, these studies demonstrate that CRISPR/dCas-based manipulation of chromatin architecture can bridge fundamental and translational research. By enabling precise control of chromatin loops, epigenetic states, enhancer activity, and nuclear positioning, 3D genome engineering provides new opportunities for mechanistic studies, disease modeling, and the development of next-generation therapeutic strategies that target genome organization itself rather than DNA sequence alone.

## 8. Challenges

Despite substantial progress in programmable genome architecture, several challenges must be addressed before these approaches can be broadly applied in experimental and therapeutic settings. Some limitations are shared with CRISPR/Cas technologies more generally, including efficient and cell-type-specific delivery, the large size of dCas-based fusion proteins, immunogenicity of Cas proteins and delivery vectors, off-target binding, and control over the duration of the induced regulatory state [47,108,109]. These issues have been extensively discussed in the context of genome and epigenome editing and are therefore not considered in detail here. Instead, we focus on challenges that arise specifically from attempts to manipulate three-dimensional genome organization, including the context dependence of architectural perturbations, productive assembly of synthetic chromatin interactions, the dynamic nature of chromosome organization, and the possibility of non-local architectural and transcriptional consequences.

The efficiency and functional outcome of 3D genome engineering are strongly influenced by cellular and genomic context. The same engineered interaction may produce different transcriptional outcomes across cell types depending on the endogenous activity of the targeted regulatory elements and the availability of cell-type-specific transcription factors and cofactors. For example, CLOuD9-mediated juxtaposition of the β-globin LCR and HBB promoter increased HBB expression in erythroid K562 cells but not in HEK293T cells [42]. Chromatin state and target accessibility provide an additional source of variability, as nucleosome occupancy can substantially restrict Cas9 and dCas9 access to target DNA [83,110,111]. The precise position of the gRNA can also determine the outcome of architectural perturbation. This is particularly evident for CTCF-dependent insulation, where the effects of dCas-based targeting depend strongly on guide positioning relative to the CTCF-binding site [62].

Recent studies demonstrating maintenance of many enhancer–promoter contacts following cohesin depletion and transcriptional activation without stable chromatin proximity further emphasize that no single architectural mechanism universally explains gene regulation [22,30,112]. Thus, the design of 3D genome-engineering experiments should consider not only sequence specificity and genomic distance, but also cell type, chromatin accessibility, regulatory activity, architectural protein occupancy, and gRNA position.

A fundamental challenge for current chromatin-looping platforms lies in the stochastic nature of bridge formation rather than in target recognition itself. Although dCas proteins can be efficiently recruited to their respective genomic loci, productive chromatin bridging competes with multiple alternative assembly pathways. Dimerization may occur before DNA binding or involve freely diffusing proteins, thereby reducing the fraction of complexes that bridge the intended genomic sites. Consequently, target occupancy alone does not necessarily translate into efficient loop formation, highlighting the need for improved spatial and temporal control of protein assembly. Quantitative models of engineered looping similarly indicate that increasing the concentration of looping components does not necessarily increase looping efficiency, as excessive concentrations can favor independent saturation of target sites rather than productive bridging [42]. Optimization of protein concentration, interaction affinity, target occupancy, and component stoichiometry is therefore important for maximizing productive loop formation as discussed in Section 6.

An additional challenge arises from the intrinsically dynamic nature of genome architecture. Chromatin loops, enhancer–promoter interactions, transcriptional hubs, and nuclear compartments are not static structures but undergo continuous formation and dissolution, resulting in substantial temporal and cell-to-cell heterogeneity in chromatin interactions [1,2]. By contrast, many current CRISPR/dCas-based platforms establish relatively persistent interactions through constitutive recruitment of architectural proteins or induced dimerization. Although such systems provide powerful tools for testing the causal role of individual chromatin interactions, they may not fully recapitulate the temporal dynamics of endogenous genome organization. The functional consequences of engineered interactions may therefore depend not only on whether a contact is formed, but also on its frequency, lifetime, and timing relative to other regulatory processes. Future systems with reversible and tunable temporal control should help distinguish effects requiring persistent architectural changes from those mediated by transient interactions. Integrating these dynamic parameters with quantitative modeling and single-cell measurements will be important for developing more predictive strategies for programmable genome architecture.

Finally, translation of chromatin engineering technologies into clinical applications faces challenges common to many CRISPR-based therapeutics. Efficient in vivo delivery of large dCas fusion proteins, multiplexed guide RNAs, and regulatory effectors remains difficult [113], while prolonged expression may increase the risks of immunogenicity and unintended effects [114]. Development of smaller programmable DNA-binding proteins, transient delivery strategies and RNA-based systems may help overcome these limitations.

Beyond these technical limitations, future progress will increasingly depend on the development of predictive frameworks capable of linking genome sequence, chromatin architecture and transcriptional output. Integration of high-resolution chromosome conformation capture, live-cell imaging, polymer modeling and artificial intelligence may ultimately transform chromatin engineering from an empirical approach into a rationally designable technology. Such advances will be essential for realizing the full potential of programmable genome architecture in both basic research and precision medicine.

## 9. Outlook and Conclusions

In little more than a decade, the study of three-dimensional genome organization has progressed from descriptive mapping toward programmable engineering. The CRISPR/dCas toolbox surveyed in this review now enables researchers to write, erase, and rewire chromatin architecture at defined loci: to establish or disrupt enhancer–promoter loops [42,43,44,45,46], tune cohesin-mediated loop extrusion through programmable barriers and epigenetic editing of architectural elements [61], reposition genomic loci between subnuclear compartments [77,80], and remodel local chromatin accessibility or seed chromatin-associated condensates [86,87]. Collectively, these approaches transform the 3D genome from a structure that can only be observed into one that can be perturbed with spatial, temporal, and increasingly combinatorial precision, enabling causal experiments that chromosome-conformation capture and imaging methods cannot provide on their own.

Beyond established dCas platforms, recently discovered programmable elements offer fundamentally different mechanisms for manipulating genome organization. Bridge RNA systems, identified in IS110-family mobile genetic elements, use a bispecific RNA molecule to pair target and donor DNA sequences simultaneously [115]. Unlike dCas-based looping systems, this natural mechanism does not depend on an additional protein–protein dimerization interface. Catalytically inactive derivatives of the IS110 recombinase–bridge RNA complex could therefore potentially serve as modular RNA-encoded scaffolds for bringing distant chromosomal sites together without modifying the underlying DNA. Similarly, TIGR–Tas systems have structural and biophysical properties that could be exploited for 3D genome engineering [116]. Whereas Cas9 forms an R-loop through guide-RNA pairing with one DNA strand and depends on PAM recognition [117], Tas uses a dual-spacer guide RNA that base-pairs with both strands of the DNA duplex [116]. Catalytically inactive Tas variants might consequently provide stable and compact programmable DNA-binding platforms or physical barriers to cohesin-mediated loop extrusion. Their small size and reported lack of a strict PAM requirement could additionally permit more flexible positioning within genomic regulatory elements, although these architectural applications remain to be experimentally demonstrated.

A major priority for the next phase of the field is overcoming the practical constraints of delivery and compactness. Common in vivo delivery vectors, such as adeno-associated viruses (AAVs), have a payload capacity of approximately 4.7 kb [113], whereas many architectural dCas constructs require large DNA-binding proteins, multiple guide RNAs, and additional effector or dimerization modules. Continued progress will therefore depend on compact, genetically encoded designs, including single-transcript guide architectures requiring only one Cas protein [46], smaller Cas orthologs, and minimal effector domains that retain architectural activity while minimizing ectopic interactions and toxicity. Recently characterized miniature DNA-targeting systems—including minimal Cas12 [118], TnpB and IscB [119], and Fanzors [120]—as well as RNA-targeting systems such as Cas13e [121,122], are generally much smaller than conventional effectors such as SpCas9. Their reduced footprint could free vector capacity for multiplexed guide arrays, regulatory modules, and cell-type-specific control elements.

A complementary strategy is to transfer part of the engineering burden from the protein to the RNA component of the complex. Guide RNA engineering can leave the protein scaffold unchanged while encoding additional functions in appended nucleotide modules [123]. Structural studies have shown that the exposed tetraloop and stem loop 2 of the Cas9 guide RNA tolerate insertions with relatively little disruption of DNA targeting [124]. Engineered guides have consequently been used to recruit transcriptional activators [125], visualize up to six genomic loci simultaneously using a single dCas9 background [126], and deliver RNA cargoes as large as 4.8 kb to selected genomic sites [127]. The latter result demonstrates the structural tolerance and modularity of the guide RNA scaffold, although large RNA cargoes would not themselves alleviate AAV packaging constraints. The delivery advantage will instead depend on the use of short RNA interaction modules that can replace substantially larger protein domains.

Guide RNA engineering could be particularly valuable for architectural applications because it provides sequence-defined orthogonality and conditional control. RNA–RNA or RNA–adapter interactions could offer a larger design space for multiplexing than the limited repertoire of protein dimerization interfaces—a constraint that has also motivated the development of exogenously supplied, selectively addressable adapters [127]. Moreover, guide RNAs can be rendered conditional by sequestering their spacer within an antisense blocker or inhibitory hairpin and releasing it through ligand binding, RNA cleavage, or toehold-mediated strand displacement [128,129,130]. Subsequent designs have decoupled the trigger sequence from the DNA target, enabling CRISPR activity to respond to unrelated endogenous transcripts [131] or endogenous miRNAs [132]. Applied to chromatin engineering, these principles suggest a still-unrealized class of tools in which two dCas-bound loci are bridged through complementary guide RNA modules and contact formation is gated by a cell-state-specific RNA signal. Their feasibility will depend on RNA-pairing stability, bridging geometry, resistance to degradation, avoidance of off-target hybridization, and sensitivity to physiological trigger concentrations.

RNA-targeting platforms provide a further opportunity to manipulate genome organization through the architectural RNAs that contribute to nuclear compartmentalization. Minimal dCas13 systems, including Cas13e, or Cas13-mediated RNA editing could be used to access, reposition, or modify nuclear RNAs without directly altering genomic DNA [121,122,133,134]. Potential targets include architectural RNAs such as *NEAT1* in paraspeckles [35], eRNAs [135], and lncRNAs that scaffold subnuclear compartments or stabilize regulatory contacts [136]. Such approaches would extend 3D genome engineering from manipulating DNA-associated proteins to directly controlling the RNA components of nuclear architecture.

Closely linked to compactness are the priorities of specificity and durability. Dimerization-based looping systems can assemble before occupying their intended genomic targets, sequestering effectors into non-productive complexes that limit looping efficiency. Bio-orthogonal and selectively addressable chemistries that connect only the intended locus pairs could provide a route to crosstalk-free multiplexing [44]. At the same time, the persistence and heritability of engineered chromatin states—rather than their mere induction—will determine whether a transient intervention produces a lasting regulatory change. For permanent architectural modifications, PASTE (Programmable Addition via Site-specific Targeting Elements) [137] and CRISPR-associated transposases [138,139] could, in principle, install synthetic CTCF-binding sites without generating double-strand breaks. Orientation-controlled insertion of these elements could establish new, cell-heritable cohesin barriers or restore disrupted TAD boundaries. However, permanent architectural editing will require particularly stringent control of insertion specificity and careful evaluation of unintended effects on neighboring regulatory domains.

A further priority is the transition from trial-and-error experimentation toward rational and predictive design. Quantitative biophysical models incorporating binding-site occupancy, protein–DNA affinity, dimerization strength, protein concentration, genomic distance, and the distance-dependent J-factor already rationalize the behavior of engineered looping complexes [42]. Extending these models with kinetic parameters, chromatin-polymer properties, and cohesin-mediated loop extrusion [20] could enable design parameters to be optimized in silico before experimental implementation. Generative machine-learning approaches point in the same direction. ProtGPS, which predicts and designs protein subcellular localization from sequence, illustrates how artificial intelligence could generate synthetic subnuclear tethering domains with prescribed localization behavior [82], while structure-based diffusion models could facilitate the design of compact architectural proteins and minimal effector domains [140]. Combined with live-cell locus imaging and quantitative measurements of loop formation, these approaches could transform 3D genome engineering into a predictive discipline. Achieving this goal will also require shared benchmarks, as most current systems have been evaluated at a single locus, in one cell type, and using one principal readout. For example, one could use a DNA biophysical model to convert the distance between two binding sites on the DNA to the J-factor, which along with other parameters (e.g., DNA binding and dimerization constants; those could be either measured in experiments or predicted with machine/deep-learning tools) could be used in a loop efficiency model to identify optimal conditions for DNA loop formation—which could simplify the implementation of the functioning system in vitro/in vivo.

Finally, the translational implications are considerable. Many diseases arise not only from coding mutations but also from altered genome topology, including developmental and imprinting disorders, laminopathies, and cancers driven by enhancer hijacking or TAD-boundary disruption. Programmable architecture engineering could therefore correct pathological gene regulation without changing the underlying coding sequence. Proof-of-concept reactivation of a silenced *MECP2* allele in Rett syndrome [69], forced regulatory looping at the globin locus [43,49], and causal repositioning of HSV-1 genomes to repress viral activity [101] already demonstrate the potential of targeted genome-architecture manipulation to restore or redirect gene regulation. Substantial obstacles remain, including efficient and cell-selective in vivo delivery, off-target chromatin effects, immunogenicity, long-term safety, and the stability of engineered states. Nevertheless, three-dimensional genome engineering is emerging as a complementary pillar to sequence-level genome editing: a strategy not only for determining which chromatin contacts are causal, but ultimately for programming genome architecture on demand.

## Figures and Tables

**Table 1:** Comparison of dCas-based strategies for engineering CTCF-dependent chromatin boundaries.

Approach	Mechanism	Reversibility	Durability/ Heritability	Specificity/Off-Target Risk	Best-Suited Application	Ref.
dCas9-DNMT3A (CBS methylation)	Epigenetic silencing of CTCF binding	Low (requires active demethylation to reverse)	High—heritable through replication	Moderate—depends on guide positioning relative to core motif	Long-term/stable boundary loss-of-function models	[61]
dCas9-KRAB/DNMT3A-DNMT3L	Combined heterochromatin + methylation	Low	High	Moderate–high, if targeted to core motif	Robust, persistent CTCF depletion	[62]
dCas9 steric interference	Direct competition at CBS	High—fully reversible	Low—requires continuous expression	High (locus-specific), but weak per-molecule effect	Acute/dynamic perturbation studies	—
Full-length CTCF recruitment	Ectopic barrier formation via native DBD	Moderate (dCas-dependent)	Moderate	Low—binds endogenous CBSs genome-wide	Proof-of-principle boundary restoration	[63,65]
N + 2 CTCF fragment	Barrier module decoupled from DNA-binding	Moderate (dCas-dependent)	Moderate	High—no endogenous CBS engagement	De novo insulation with minimal off-target effects	[65]

**Table 2:** Disease-relevant applications of CRISPR/dCas-based 3D genome engineering.

Disease/Application	Target	Tool	Model	Demonstrated Outcome	Status/Limitation
Rett syndrome	MECP2	dCas9-TET1 + dCpf1-CTCF	RTT neurons	MECP2 reactivation + improved neuronal phenotype	ex vivo proof-of-concept
Hemoglobinopathies	β-globin locus	CLOuD9	K562	altered globin expression	disease relevance inferred
HSV-1	viral genome	CRISPR-nuPin	infected cells	nuclear-periphery repositioning, reduced replication	proof-of-concept
Cancer	CTCF/TAD boundaries	epigenetic/CTCF tools	mostly mechanistic models	restoration/disruption of insulation	proposed therapeutic application

## Data Availability

No new data were created or analyzed in this study. Data sharing is not applicable to this article.

## Supplementary Materials

The following supporting information can be downloaded at: https://www.mdpi.com/article/10.3390/ijms27188104/s1.
